# Recombinant AhpC antigen from *Mycobacterium bovis* boosts BCG-primed immunity in mice

**DOI:** 10.3906/biy-2108-41

**Published:** 2021-11-14

**Authors:** Özgün Fırat DÜZENLİ, Sezer OKAY, İnci KAZKAYASI, Ayşe Filiz ÖNER

**Affiliations:** 1 Department of Pharmaceutical Biotechnology, Faculty of Pharmacy, Hacettepe University, Ankara, Turkey; 2 Department of Vaccine Technology, Vaccine Institute, Hacettepe University, Ankara, Turkey; 3 Department of Pharmacology, Faculty of Pharmacy, Hacettepe University, Ankara, Turkey

**Keywords:** Subunit vaccine, tuberculosis, prime-boost vaccination, recombinant protein

## Abstract

Tuberculosis (TB) is still one of the most common infectious diseases around the world despite the widespread use of BCG (bacille Calmette-Guerin) strain of *Mycobacterium bovis* as a vaccine. This vaccine does not always protect people from TB, and, thus, new effective vaccines or vaccination strategies are being investigated. In this study, alkyl hydroperoxide reductase (AhpC) from *M. bovis* was evaluated as a new candidate vaccine antigen against TB in BALB/c mice model. The *ahpC* gene was amplified from *M.bovis* genome, cloned, and expressed in *Escherichia coli*. Vaccine antigen AhpC was formulated with Montanide ISA 61 VG, an oil-based emulsion adjuvant. Both IgG and IL-12 responses were observed in mice after administering the formulation both as a subunit vaccine alone and also as a booster vaccine for BCG immunization. However, a long-lasting response was observed when AhpC formulation was used as a booster (for BCG-primed immunization) as compared to being used as a subunit vaccine alone. In short, these findings suggested that AhpC has the potential to be used as a booster vaccine candidate for BCG-primed immunization.

## 1. Introduction

Tuberculosis (TB) is an infectious disease that is primarily caused by the inhalation of particles containing bacilli within the *Mycobacterium tuberculosis* complex (MTBC) (Kanipe and Palmer, 2020). While *M. tuberculosis* mainly infects the human host, the primary causative agent for TB in the family *Bovidae* is *M. bovis* (Rodriguez-Campos et al., 2014). The susceptibility of humans to TB caused by *M. bovis* is attributed to its zoonotic character (Michel et al., 2010). In addition to person-to-person transmission (Evans et al., 2007; Sunder et al., 2009)., *M. bovis* is also transmitted in humans by consumption of unpasteurized or contaminated dairy products or inhalation of aerosols containing bacillus (Grange, 2001).

Since 1931, *M. bovis* Bacillus Calmette-Guérin (BCG) vaccine has been used as the only licensed vaccine against TB. Although BCG vaccine is widely used worldwide, high variability in its efficacy (0%–80%), ineffectiveness against pulmonary TB in adults (Colditz, 1994), and safety risks due to the possibility of mutation to its virulent form (Fatima et al., 2020) clearly demonstrate the need for a more effective vaccine protecting against all forms of TB in all age groups. Currently, several vaccines are under clinical trials either as potential alternatives for BCG or as booster vaccines (Whitlow et al., 2020).

Data obtained from virulence gene identification studies play an essential role both in the discovery of new drug targets and in the development of novel TB vaccine candidates. The virulence factor proteins of *M. bovis* inhibit the macrophages’ antimicrobial attacks and enhances the resistance of the bacilli against the first immune attack of the macrophages (via oxidative and nitrosative stress responses, phagosome arresting, and apoptosis inhibition) (Forrellad et al., 2013). A typical example of this is alkyl hydroperoxide reductase C (Rv2428, AhpC), a member of the peroxiredoxin family, that catalyzes the detoxification reaction of organic peroxides into less reactive derivatives. Thus, AhpC can protect the microbial pathogen against both oxidative and nitrosative stresses (Echeverria-Valencia et al., 2018).

Sequence similarity of *ahpC* genes from different *Mycobacterium* species, including *M*. *bovis*, *M*. *tuberculosis, M*. *ulcerans*, *M*. *africanum*, *M*. *smegmatis*, *M*. *sinense*, and *M*. *leprae* was shown in the paper of Wong et al. (2013). The protective effect of AhpC against oxidative stress responses of the host immune cells and correlation between ahpC gene expression and bacterial virulence were extensively investigated by other researchers (Wilson and Collins, 1996; Wilson et al., 1998). Verma et al. (2021) revealed *kasA* and a*hpC* genes as potential drug targets due to their roles in drug resistance. Considering these features of the AhpC, it was thought that it could be both a vaccine and also a drug candidate.

In this study, *ahpC* gene was amplified from *M.bovis* and heterologously produced in *E.coli* BL21(DE3) expression system. Then, purified recombinant AhpC protein was formulated with an oil-based adjuvant. We investigated the immunostimulatory effect of the antioxidant enzyme AhpC both as a subunit vaccine alone and a booster vaccine after BCG prime immunization in BALB/c mice groups.

## 2. Materials and methods

### 2.1. Bacterial strains, media, and plasmids

*Escherichia coli* strains DH5α (Novagen, Germany) and BL21 (DE3) (ATCC, USA) were used for cloning and expression of the gene *ahpC*, respectively. LB Broth with agar (Miller) and LB Broth (Miller) media were used for the cultivation of *E.coli* strains. Isolated and purified genomic DNA of *M. bovis* (ATCC 35743, GenBank CP003494.1, USA) was kindly provided by Assoc. Prof. Dr. Alpaslan Alp (Hacettepe University Faculty of Medicine, Department of Medical Microbiology, Ankara, Turkey). The pGEMT Easy (Promega, USA) and pET28a(+) (Novagen, Germany) plasmids were used for gene cloning and recombinant protein expression, respectively. Protino Ni-TED kit (Macherey-Nagel, Germany) was used to purify His-tagged recombinant protein.

### 2.2. PCR amplification of *ahpC* gene

The genomic DNA of *M. bovis* was used as the template for amplification of *ahpC* gene (588 bp). The nucleotide sequence corresponding to *ahpC* gene was amplified using polymerase chain reaction (PCR) with the following two pairs of gene-specific primers, *ahpC*F: 5′–ggatccatgccactgctaaccattg-3′ (*BamH*I site underlined) and *ahpC*R: 5′-aagcttggccgaagccttgag - 3′ (*Hind*III site underlined). All chemical and biological reagents were purchased from ThermoScientific. PCR mix composed of 2.5 mM MgCl_2_, 0,2 μM of primers, 1 ng template DNA, 0,6 mM dNTP mix (Cat. No. R0192), 1 × *Taq* buffer (with KCl, without MgCl_2_) and 0,1 U *Taq* DNA polymerase (Cat. No. EP402) was prepared in a total volume of 25 μL. The cycling program was set as 94 °C for 10 min, 30 cycles of amplification (94 °C for 1 min, 52 °C for 30 s, 72 °C for 1 min), and at 72°C for 10 min. PCR products were analyzed by electrophoresis using 1% (w/v) agarose gel.

### 2.3. Construction of recombinant plasmids

pGEMT Easy Vector System (Promega, WI, USA) was used for the ligation of PCR products. The ligation reaction was performed according to the instructions of the manufacturer, and plasmids transformed into *E. coli* DH5α. The pGEMT-*ahpC* product was verified by doubledigestion with *BamH*I and *Hind*III enzymes. Afterward, *ahpC* gene was cloned in multiple cloning site between *BamH*I and *Hind*III in expression plasmid, pET-28a (+), which encodes for the 6xHis tag. The resulting recombinant plasmid was named pET-*ahpC* and was introduced in *E.coli* DH5α. The recombinant bacteria were screened via restriction enzyme digestion of plasmids and PCR**.** pET-*ahpC* was sequenced by Sanger sequence analysis method (Sentebiolab, Turkey)

(Supplemental information). pET-*ahpC* was subsequently transformed into the host *E. coli* BL21(DE3) competent cells (Novagen) for recombinant protein expression.

### 2.4. Expression and purification of recombinant AhpC

Expression and purification of recombinant AhpC (rAhpC) were performed as described by Okay et al., (2012). *E. coli* BL21 (DE3) cells carrying pET-*ahpC* was grown in Luria Broth (LB; Merck, Germany) supplemented with kanamycin (30 μg/mL). When OD_600_ value of culture reached 0.5, isopropyl-β-D-galactopyranoside (IPTG; Sigma, Germany) was added to induce recombinant protein expression (1 mM final concentration). Incubation was carried out at 37 °C for 4 h in a shaker incubator at 200 rpm. Expression host cells were collected by centrifugation (5000 g for 5 min, at 4 °C). Subsequently, the harvested cells were resuspended in LEW buffer (Lysis-Equilibration-Washing buffer; 50 mM NaH_2_PO_4_, 300 mM NaCl, 8 M urea, pH 8.0). Next, cells were lysed using an ultrasonic probe (Bandelin-Sonoplus, Germany) at 60% amplitude, 10 s pulses at six intervals. Cellular debris was removed by centrifugation, and the supernatant containing recombinant protein was collected. Purification of the protein was performed by applying the supernatant to a Ni-NTA affinity column (Protino Ni-TED 2000 packed columns, Macherey–Nagel, Germany) according to the supplier’s instructions. Eluted proteins were concentrated by Amicon ultrafiltration device (Merckmillipore, USA), sterilized through a 0.2 μm membrane filter, and stored at −20 °C until use. Bradford’s method (1976) was used to quantify the recombinant protein.

### 2.5. Characterization of recombinant AhpC

The protein was further subjected to sodium dodecyl sulfate-polyacrylamide gel electrophoresis (SDS–PAGE) and Western-blotting to analyze the expression. Briefly, a purified protein sample was run at the rate of 4%–12% SDS-polyacrylamide gels (Laemmli, 1970). Coomassie Blue R-250 staining protocol was performed for one of the gels, and the other was transferred to nitrocellulose membrane (0.45 μm) by processing via a modified Towbin method (1979). The anti rAhpC antibody obtained from the 60^th^-day serum of mice vaccinated with rAhpC, at a dilution factor of 1:400 (v/v) was used as the primary antibody. Alkaline phosphatase (AP)-conjugated antimouse IgG (Sigma, Germany) was used as the secondary antibody (at a dilution of 1:20.000 (v/v)). To visualize protein bands on nitrocellulose membrane, AP Conjugate Substrate Kit (Bio-Rad, CA, USA) was applied.

### 2.6. Preparation of vaccine formulations

A 1 mg/mL rAhpC stock solution was prepared in PBS. The antigen (rAhpC) solution and adjuvant (Montanide 61VG, Seppic) components were mixed at a ratio of 2:3 (v/v) under aseptic conditions by vortexing for 2 mins. Prior to use, the sterility of prepared vaccine formulations was tested using aerobic culture on LB-agar incubated at 37 °C for 48 h.

### 2.7. Animals and vaccination

6–8 week old female BALB/c mice were immunized in animal experiments. Animal experiments were performed under the approval of the Ethics Committee on Animal Experimentation, Hacettepe University, Turkey (No: 2020/08-16). Animals were immunized subcutaneously in groups of six with one of the following treatment conditions:

-Group A (n = 6): Adjuvant control group; immunized with 250 μL PBS – ISA 61VG mixture (2:3 v/v, administrated at day 0 and 15).
-Group B (n = 6): BCG control group; immunized with 0.1 mL, 5×10^6^ CFU BCG vaccine (Serum Institute of India), given once (at day 0) and injected with 250 μL of PBS two times (administrated at day 15 and 30).
-Group C (n = 6): BCG Prime – ISA 61VG Boost group; immunized with 0.1 mL prime BCG with 5×10^6^ CFU, given once (at day 0) and boosted with 250 μL total volume of ISA 61VG (administrated at day 15 and 30).
-Group D (n = 6): Adjuvanted rAhpC group; immunized with 250 μL purified rAhpC formulated with ISA 61VG (2:3, v/v, administrated at day 0 and 15).
-Group E (n = 6): BCG Prime – AhpC Boost group; immunized with 0.1mL prime BCG (5×10^6^ CFU), given once (at day 0) and boosted with 250 μL purified rAhpC formulated with ISA 61VG (2:3, v/v, administrated at day 15 and 30).

**Figure 1:**
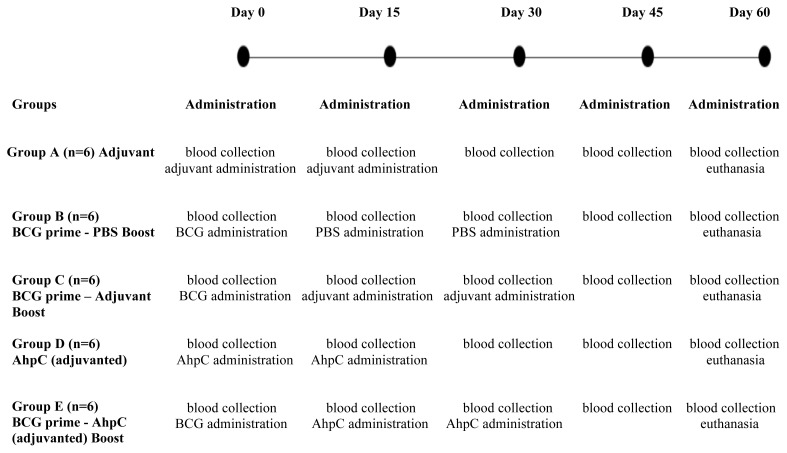
Schematic illustration of immunization schedule. Blood samples were collected just before vaccine administration at days 0, 15, and 30. ***Abbr.*** PBS, phosphate buffered saline.

The tail vein of mice was chosen for the collection of blood samples at day 0, 15, 30, 45, and 60. The collected serum samples were stored at –20 °C until use. Mice were euthanized via cervical dislocation at day 60. The immunization schedule and blood collection times of the groups are illustrated in Figure 1.

### 2.8. Detection of antibody response

AhpC-specific IgG levels were measured by enzyme-linked immunosorbent assay (ELISA). 96-well plates were coated with rAhpC protein (1 μg/well). Sera collected from vaccinated mice were used as the primary antibody. Two-fold serial dilutions of primary antibodies (from 1:50 to 1:6400, v/v) were applied in plates in duplicates. Alkaline phosphatase-conjugated anti-mouse IgG (Sigma, USA) was used as a secondary antibody at a dilution factor of 1:1000 (v/v). The AP Conjugate Substrate Kit was used as a colorimetric reagent (Bio-Rad, USA). Optical density was measured at 405 nm.

### 2.9. IL-12 assay

Mouse IL-12 ELISA Total Kit (Thermo Scientific) was used according to the supplier’s instructions to measure the cellular immune response in the vaccinated mice. The sera collected from the vaccinated mice were used as the primary antibody, and the level of serum IL-12 was calculated via a standard curve.

### 2.10. Statistical analysis

ELISA data were analyzed by using the Graphpad Prism 8 software using two-way analysis of variance (ANOVA) and a posthoc test (Tukey’s test). P values *<* 0.05 were considered as significant.

## 3. Results

### 3.1. Cloning of *ahpC* gene

**Figure 2:**
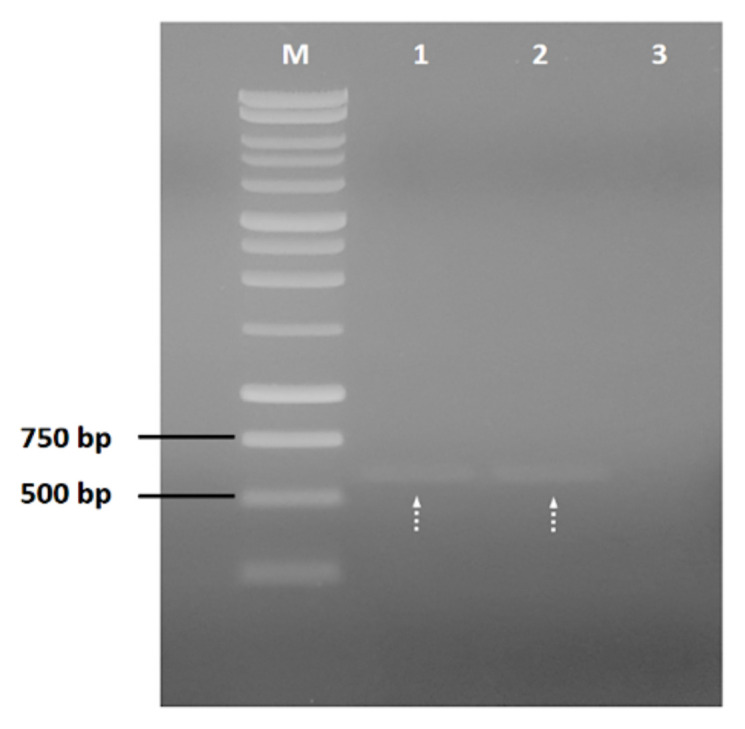
Agarose gel image of *ahpC* gene was amplified by PCR from *M.bovis* genome. M: DNA marker (G571A, Promega); 1,2: PCR amplified *ahpC*; 3: Negative control PCR tube. Dashed arrows show the expected amplicon size for the AhpC gene (588 bp, GenBank CP003494.1, location: 2400625 .. 2401212 ).

The *ahpC* gene from *M. bovis* (588 bp, GenBank CP003494.1, location: 2400625 .. 2401212) was successfully amplified (Figure 2). Subsequently, the *ahpC* gene was cloned in pGEM-T Easy vector system and pET-28a(+) for gene amplification and recombinant protein purification, respectively.

### 3.2. SDS-PAGE and Western blot analyses of rAhpC protein

**Figure 3:**
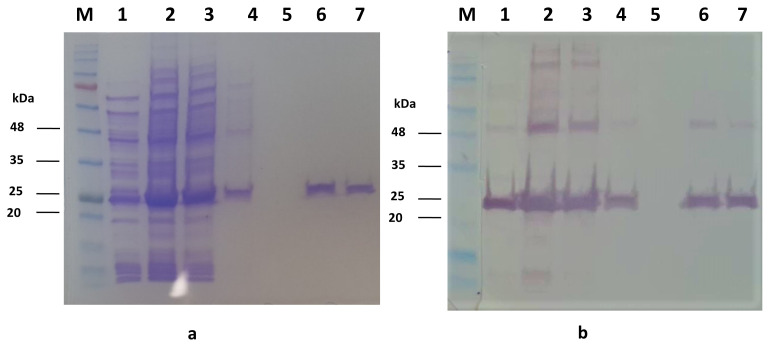
Gel images of SDS-PAGE analyis by Coomassie blue staining **(A)** and Western blot **(B)**. The primary antibodies used in WB were obtained from the 60th day serum of AhpC vaccination group (Group D). M: Prestained protein ladder (Bio-Helix, PM007), 1: Lysate of *E.coli* carrying pET-*ahpC* (IPTG non-induced), 2: Lysate of *E.coli* carrying pET-*ahpC* (IPTG-induced), 3: Flow-through fraction, 4–5: First and second washing fractions, respectively, 6–7: First and second elutions of recombinant AhpC protein, respectively.

pET-28a(+) vector encoding *ahpC* was transformed into *E.coli* BL21(DE3) for rAhpC production. rAhpC produced in E.coli was purified using nickel columns. The molecular weight (MW) of the rAhpC (including His-tags) was predicted as 22.389 kDa using a web-based tool (ProtParam, https://web.expasy.org/protparam). The observed MW of the rAhpC protein on SDS-PAGE was approximately 25 kDa (Figure 3a). Protein-specific sera were used as primary antibody in Western blot analysis. Antibodies in sera also bind ~50 kDa dimerized rAhpC protein (Figure 3b).

### 3.3. Humoral immune response against AhpC

Antibody-mediated immune responses depend on different functions of multiple classes of antibodies (i.e, IgM, IgA, IgG, and IgE). Among them, IgG is known with its antigen specific high affinity and also its ability for neutralization of the infectious pathogens as well as its Fc-mediated effector functions. On the other hand, its characteristics such as abundance and long half-life time in blood and also interaction with differentiated memory B cells render immunoglobulin G as a good indicator for humoral immune responses (Galipeau et al., 2020).

**Figure 4:**
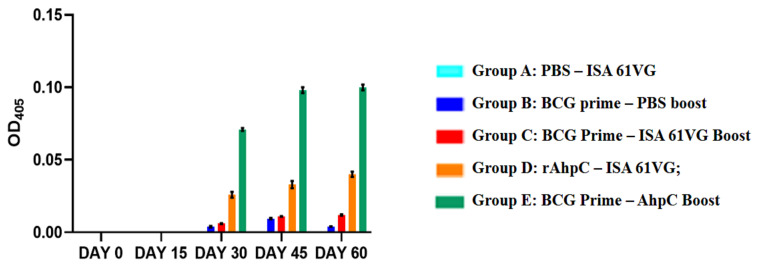
Measured OD_405_ values of total serum IgG in BALB/c mice. Samples were diluted by a dilution factor of 1:200. PBS was used as blank solution, and measured OD_405_ value of PBS has been subtracted from all absorbances. ***Abbr.*** PBS; phosphate buffered saline. Group A: PBS – ISA 61VG (turquoise blue); Group B: BCG prime – PBS boost (Navy blue); Group C: BCG Prime – ISA 61VG Boost (red); Group D: rAhpC – ISA 61VG (orange); Group E: BCG Prime – AhpC Boost (green).

In this study, quantitative detection of the humoral immune response against AhpC protein was tested using ELISA method. The total IgG level in collected sera of mice groups was evaluated at day 0, 15, 30, 45, and 60 (Figure 4) using two-way ANOVA and Tukey’s test (Table 1).

All vaccination regimens were well tolerated by the mice groups. In Group A (adjuvant ISA 61 VG vaccination group), the anti-AhpC IgG was not detected until day 60. All other groups, on the other hand, showed a AhpC specific IgG response from day 30 indicating that BCG or adjuvanted AhpC vaccination requires at least 30 days to induce anti-AhpC antibodies. Introduction of second second dose adjuvanted AhpC elicited antibody production in Group D (adjuvanted rAhpC group), but it was not as high as in Group E (BCG prime – AhpC boost group). At day 45 and 60, the serum of mice in Group C (BCG Prime – ISA 61VG boost group) or Group B (BCG control Group) retained anti-AhpC IgG antibodies. Likewise, at day 45 and 60, increased anti-AhpC IgG levels were detected in Group D (Adjuvanted AhpC group) and Group E (BCG prime – AhpC boost group). The results indicate that administering the second dose of AhpC as a booster increases the anti-AhpC level up to 60 days (Figure 4).

Our results showed that vaccination with adjuvanted AhpC alone was more effective than ISA 61 VG alone (Group A vaccination group) and BCG alone (Group B vaccination group) at the end of 60 days (p < 0.001 and p < 0.01, respectively, Table 1). However, when adjuvanted AhpC was used as a booster to prime BCG vaccine, a more potent and sustainable humoral immune response was induced during the 60-day period (Figure 4 Table 1).

In Group E, single-dose adjuvanted AhpC vaccination after BCG prime immunization induced a stronger humoral response compared to Group D, which was immunized with two doses of adjuvanted AhpC (p < 0.001, Figure 4, Table 1). Moreover, injection of a second booster dose of adjuvanted AhpC in Group E increased the serum antibody level more (at day 45).

In a nutshell, anti-AhpC IgG antibodies were induced both with AhpC vaccine alone and BCG primed – AhpC booster vaccine regimens at day 60.

### 3.4. Cellular immune responses induced by vaccinations

**Figure 5:**
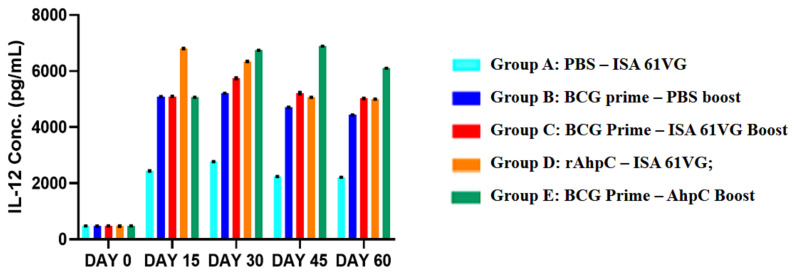
Serum IL-12 concentrations of BALB/c mice at different time intervals. **Abbr.** Conc; concentration. Group A: PBS – ISA 61VG (turquoise blue); Group B: BCG prime – PBS boost (Navy blue); Group C: BCG Prime – ISA 61VG Boost (red); Group D: rAhpC – ISA 61VG (orange); Group E: BCG Prime – AhpC Boost (green).

The change in serum IL-12 level in the vaccination groups at day 0, 15, 30, 45, and 60 (Figure 5) was evaluated (Table 2).

In all mice groups, the srum IL-12 was at basal level at day 0. At day 15, an increase in serum IL-12 level was observed in all groups with the highest value measured in adjuvanted AhpC treated group. The adjuvant effect of booster ISA 61 VG is clearly witnessed (at day 30, 45 and 60) in the differences in serum IL-12 level between Group B (BCG control group) and Group C (BCG prime – ISA 61 VG boost group). The highest IL-12 levels were achieved in Group E after first AhpC bosster as well as after second AhpC booster. At day 60, a decrease in serum IL-12 was observed in all groups, yet still, the highest serum IL-12 level was measured in Group E (BCG prime – AhpC bosst group) (Figure 5).

At day 15, although an increase in serum IL-12 level was observed in all groups after the first vaccination, the highest titer was realised in adjuvanted AhpC vaccination group (Group D). On the contrary, an increase in serum IL-12 titers was seen in all groups except adjuvanted AhpC vaccination group (Group D) at day 30. Interestingly, a second booster dose of adjuvanted AhpC led to a decrease in serum IL-12 concentration, unlike the first booster dose, which raised the IL-12 levels. At days 30, 45, and 60, Group C (BCG prime – ISA 61 VG boost group) had relatively higher levels of IL-12 as compared to Group B (BCG control group) (p < 0.01, p < 0.001 and p < 0.0001, respectively) due to the adjuvant effect of Montanide ISA 61 VG. At days 45 and 60, the serum IL-12 level in adjuvanted AhpC vaccination (Group D) was less than Group E (BCG prime – AhpC boost group).

In summary, adjuvanted AhpC did not elicit strong immune responses when it was administrated alone; however, an immunostimulation enhancer effect was observed when it was used as a booster vaccine in BCG prime immunized mice.

## 4. Discussion

Currently, BCG is widely used in many countries in the childhood vaccination program to prevent severe forms of TB in children. However, BCG vaccination is considered not sufficient aginst TB infection in adults (World Health Organization, 2020). Therefore, efforts in the prevention of TB are mainly focused on the development of new vaccines, new drugs, or innovative treatment strategies. Currently, various new drugs, vaccines, and combination regimens are under investigation in clinical trials.

In this study, a new recombinant vaccine formulation composed of AhpC and an oil-based adjuvant Montanide ISA 61 VG was administered to mice as a subunit vaccine alone and as a booster after BCG-prime vaccination.

Although AhpC is a protein with a molecular wight of ~25 kDa, we observed two distinct bands (~25 kDa and ~50 kDa) in SDS-PAGE as shown in the study of O’Riordan et al. (2012). The band of 50 kDa was due to the possible dimerization of AhpC between two cysteine sulfhydryls in protein structure (Hillas et al., 2000; Chauhan and Mande, 2002). Two bands at 25 and 50 kDa were also detected in Western blot, which belongs to AhpC and its dimer form, respectively. Although this lane was lysate of non-IPTG-induced *E.coli* BL21(DE3) harboring pET-*ahpC*, the protein bands were probably a result of phenomenon known as the leaky expression of T7promoter – T7RNA polymerase system (McCutcheon, 2018).

Disperse systems, chemical or biological molecules can be used as adjuvants in order to enhance the immunogenicity of an antigen. Studies have been conducted to evaluate adjuvant’s potential use in the vacines against infectious diseases, cancer, and autoimmune diseases (Shah et al., 2015). Montanide adjuvant system includes mineral or non-mineral oils, a mannitol-based surfactant, and purified oleic acid from vegetable origin (Jang et al., 2010). They are classified as emulsions, micro-emulsions, and polymeric gels according to their preparation technology (Seppic, 2017). Montanide ISA 61 VG adjuvant is a readyto-use mineral oil-based stable w/o emulsion formulation, and it induces high-level and long-lasting immune responses in animals (Khorasani et al., 2016).

Since subunit vaccines contain only the antigenic parts of the pathogen, replication in the host is not possible. Therefore, they have advantages in terms of safety considerations. On the other hand, certain drawbacks such as the requirement of multiple doses and coadministration of adjuvant(s) to elicit a vigorous humoral or cellular immune response against the antigen(s) of interest hinders the use of vaccines (Hansson et al., 2000). In this study, no anti-AhpC antibodies were detected in the adjuvanted AhpC vaccination group (Group D) 15 days after the first dose, but, rather, the anti-AhpC antibodies were detected after the second dose adjuvanted AhpC administration (boost injection). At day 60 of the study, anti-AhpC antibodies reached maximum levels 4 weeks after booster injection, also reported by O’Riordan et al. (2012). Although more studies are required for a better understanding of the mechanism of action of adjuvants used in vaccine formulations, it is believed that water-in-oil emulsion-based adjuvants induce local inflammation and increase the recruitment and activation of antigenpresenting cells (Leroux-Roels, 2010). Enhancer effect of ISA 61 VG adjuvant on antibody responses was reported by others (İz et al., 2018). We observed this effect also in our study by comparing the results of Group B (BCG control group) and Group C (BCG prime – ISA 61 VG boost group). First and second booster doses of ISA 61 VG given to BCG-prime vaccinated mice enhanced the production of anti-AhpC IgG at day 30 and 45, respectively (Table 1, p < 0.05). At the end of immunization (day 60), serum anti-AhpC antibody level of Group C was higher than Group B (Table 1, p < 0.001).

The importance of cell-mediated responses for immune protection against intracellular pathogens such as *M. tuberculosis* is well known. Cytokines such as interferon-γ (IFN-γ), tumor necrosis factor α (TNF-α), and interleukin 12 (IL-12) play a vital role in protective cell-mediated immune response against TB disease (Kaufmann, 2008). Therefore, the selection of appropriate adjuvant is a critical step for subunit vaccine formulations, since either humoral or cellular immune responses can be induced depending on the type of adjuvant used in the vaccine formulation. In addition to stimulating the antibody responses, Montanide ISA 61 VG adjuvant also has a strong inducer effect on cellular immune response (Ibrahim et al., 2015). Our observation of serum IL-12 levels of the mice groups revealed an outcome that is different from the results of IgG responses. Unlike with the antibody response, which constantly increased up to day 60, the serum IL-12 level decreased after second booster doses at day 30 (only for Group D), day 45 (for all groups except for Group E), and day 60 (for all groups). This observation could be attributed to a negative feedback loop, due to the release of immunoregulatory cytokines or imbalance between regulatory and effector T cell subsets, as suggested by Begg et al. (2019). Nevertheless, the rise in serum IL-12 level in Group A demonstrates the cellular immune response enhancing ability of the adjuvant. Between Group B and Group C, the latter had relatively higher levels of serum IL-12 at days 30, 45, and 60.

A study conducted by Karonga Prevention Trial Group to evaluate the effect of revaccination with BCG in humans has revealed that a second BCG vaccination could not provide any protection against tuberculosis. It was suggested that boosting of immune responses in BCG-primed vaccinations could possibly enhance BCG immunity (Valdés et al., 2019). In this light, Yang et al. (2016) showed in their study that the use of BCG prime – subunit recombinant protein vaccine immunization strategy could enhance the cellular immune response in mice. Different boosting strategies and the importance of prime – boost immunization in vaccination against TB were well reviewed by Dalmia and Ramsay (2012). Overall, immune response against *M. tuberculosis* by a two-stage vaccination regimen based on priming with BCG and boosting with the most effective subunits seems to be the most promising approach. When the effect of adjuvanted AhpC used as subunit vaccine alone was compared with AhpC used as a booster to BCG prime in BALB/c mice, it was realised that a single dose adjuvanted AhpC could not induce anti-AhpC antibody response unless supplemented with a second dose booster. However, a single dose AhpC as a booster after BCG – prime vaccination elicited higher IgG responses.

When it comes to cellular immune response, it was observed that group treated with a single dose of adjuvanted AhpC induced a stronger IL-12 response as compared to adjuvant and BCG vaccination groups. But afterwards, second dose adjuvanted AhpC administration caused a decrease in serum IL-12 level. Based on this observation, adjuvanted AhpC vaccination could be said to induce the highest level of IL-12. However, a more robust profile for strong humoral responses was obtained in BCG prime – AhpC boost vaccination group (Group E), suggesting that ‘BCG prime – AhpC boost’ vaccination could be a useful strategy for the development of a TB vaccine against tuberculosis disease. Further bacterial challenge studies are needed to evaluate the immunoprotective effect of BCG prime – AhpC boost vaccination strategy against tuberculosis disease caused by virulence strain of *M. bovis* or *M. tuberculosis*.

In this study, we evaluated the effect of antioxidant enzyme, AhpC, on humoral and cellular immune responses in BALB/c mice for a period of 60 days. A new recombinant vaccine formulation composed of AhpC protein antigen, and an oil-based adjuvant Montanide ISA 61 VG was administered to mice as a subunit vaccine formulation alone and as a booster for BCG – prime vaccination. Based on our results, the BCG prime – AhpC boost vaccination strategy prolonged both humoral and cellular immune response. It can be concluded that AhpC, an antioxidant protein, is a promising subunit vaccine antigen when used in BCG prime-AhpC protein boost vaccination strategy against TB.

## Supplemental Information

### Sanger Sequence Analysis of pET-*ahpC*

GGCCCTTCCCTCCTCCAGCTCACCGCTCTCATCGGCGGTGACCTGTCCAAGGTCGACGCCA AGCAGCCCGGCGACTACTTCACCACTATCACCAGTGACGAACACCCAGGCAAGTGGCGG GTGGTGTTCTTTTGGCCGAAAGACTTCACGTTCGTGTGCCCTACCGAGATCGCGGCGTTCA GCAAGCTCAATGACGAGTTCGAGGACCGCGACGCCCAGATCCTGGGGGTTTCGATTGACA GCGAATTCGCGCATTTCCAGTGGCGTGCACAGCACAACGACCTCAAAACGTTACCCTTCC CGATGCTCTCCGACATCAAGCGCGAACTCAGCCAAGCCGCAGGTATCCTCAACGCCGACG GTGTGGCCGACCGCGTGACCTTTATCGTCGACCCCAACAACGAGATCCAGTTCGTCTCGG CCACCGCCGGTTCGGTGGGACGCAACGTCGATGAGGTACTGCGAGTGCTCGACGCCCTCC AGTCCGACGAGCTGTGCGCATGCAACTGGCGCAAGGGCGACCCGACGCTAGACGCTGGC GAACTCCTCAAGGCTTCGGCCAAGCTTGCGGCCGCACTCGAGCACCACCACCACCACCAC TGAGATCCGGCTGCTAACAAAGCCCGAAAGGAAGCTGAGTTGGCTGCTGCCACCGCTGA GCAATAAACTAGCATAACCCCTTGGGGCCTCTAAACGGGTCTTGAGGGGTTTTTTGCTGA AAGGAGGAACTATATCCGGATTGGCGATGGGGACGCGCCATGTAACGGCGCTATAAACG CGGCAGGTGT

### BLAST analysis (https://blast.ncbi.nlm.nih.gov/Blast.cgi#1765) of Sanger sequenced pET-*ahpC*





## Figures and Tables

**Table 1:** Analysis of variance (ANOVA) for serum IgG levels in BALB/c mice groups.

Vaccination Groups	Day 0	Day 15	Day 30	Day 45	Day 60
Group A – Group B	NS	NS	*	***	****
Group A – Group C	NS	NS	**	****	****
Group A – Group D	NS	NS	**	**	***
Group A – Group E	NS	NS	****	****	****
Group B – Group C	NS	NS	*	*	***
Group B – Group D	NS	NS	**	*	**
Group B – Group E	NS	NS	****	****	****
Group C – Group D	NS	NS	**	*	**
Group C – Group E	NS	NS	****	****	****
Group D – Group E	NS	NS	***	****	****

*Note:* PBS measurements were subtracted from those of vaccination groups. Day 0 is considered as the point of pre-immunization.

* p < 0.05.

** p < 0.01.

*** p < 0.001.

**** p < 0.0001;

NS: not significant.

*Group A: PBS – ISA 61VG (turquoise blue); Group B: BCG prime – PBS boost (Navy blue); Group C: BCG Prime – ISA 61VG Boost (red); Group D: rAhpC – ISA 61VG (orange); Group E: BCG Prime – AhpC Boost (green)*.

**Table 2:** Analysis of variance (ANOVA) for serum IL-12 levels in BALB/c mice groups.

Vaccination Groups	Day 0	Day 15	Day 30	Day 45	Day 60
Group A – Group B	NS	****	****	****	****
Group A – Group C	NS	****	****	****	****
Group A – Group D	NS	****	****	****	****
Group A – Group E	NS	****	****	****	****
Group B – Group C	NS	NS	**	**	****
Group B – Group D	NS	****	****	****	****
Group B – Group E	NS	NS	****	****	****
Group C – Group D	NS	****	***	*	NS
Group C – Group E	NS	NS	****	****	****
Group D – Group E	NS	****	***	****	****

*Note:* Day 0 is considered as the time point of pre-immunization.

* p < 0.05.

** p < 0.01.

*** p < 0.001.

**** p < 0.0001;

NS: not significant.

*Group A: PBS – ISA 61VG (turquoise blue); Group B: BCG prime – PBS boost (Navy blue); Group C: BCG Prime – ISA 61VG Boost (red); Group D: rAhpC – ISA 61VG (orange); Group E: BCG Prime – AhpC Boost (green).*

## References

[b1-turkjbiol-46-1-95] Begg DJ Dhungyel O Naddi A Dhand NK Plain KM 2019 The immunogenicity and tissue reactivity of *Mycobacterium avium subsp paratuberculosis* inactivated whole cell vaccine is dependent on the adjuvant used Heliyon 5 e01911 10.1016/j.heliyon.2019.e01911 31249894 PMC6584770

[b2-turkjbiol-46-1-95] Bradford MM 1976 A rapid and sensitive method for the quantitation of microgramquantities of protein utilizing the principle of protein-dye binding Analytical Biochemistry 72 248 254 10.1016/0003-2697(76)90527-3 942051

[b3-turkjbiol-46-1-95] Chauhan R Mande SC 2002 Site-directed mutagenesis reveals a novel catalytic mechanism of *Mycobacterium tuberculosis* alkylhydroperoxidase C Biochemical Journal 367 1 255 261 10.1042/bj20020545 12084012 PMC1222857

[b4-turkjbiol-46-1-95] Colditz GA Brewer TF Berkey CS Wilson ME Burdick E 1994 Efficacy of BCG vaccine in the prevention of tuberculosis: Meta-analysis of the published literature JAMA 271 9 698 702 10.1001/jama.1994.03510330076038 8309034

[b5-turkjbiol-46-1-95] Dalmia N Ramsay AJ 2012 Prime-boost approaches to tuberculosis vaccine development Expert Review of Vaccines 11 10 1221 1233 10.1586/erv.12.94. 23176655 PMC3572762

[b6-turkjbiol-46-1-95] Echeverria-Valencia G Flores-Villalva S Espitia CI 2018 Virulence factors and pathogenicity of Mycobacterium Ribón W Mycobacterium - Research and Development London: United Kigdom InTech 231 255

[b7-turkjbiol-46-1-95] Evans JT Smith EG Banerjee A Smith RMM Dale J 2007 Cluster of human tuberculosis caused by *Mycobacterium bovis*: evidence for person-to-person transmission in the UK The Lancet 369 1270 1276 10.1016/S0140-6736(07)60598-4 17434402

[b8-turkjbiol-46-1-95] Fatima S Kumari A Das G Dwivedi VP 2020 Tuberculosis vaccine: A journey from BCG to present Life Sciences 252 117594 10.1016/j.lfs.2020.117594 32305522

[b9-turkjbiol-46-1-95] Forrellad MA Klepp LI Gioffré Ay García JS Morbidoni HR 2013 Virulence factors of the *Mycobacterium tuberculosis* complex Virulence 4 1 3 66 10.4161/viru.22329 23076359 PMC3544749

[b10-turkjbiol-46-1-95] Grange JM 2001 *Mycobacterium bovis* infection in human beings Tuberculosis 81 1/2 71 77 10.1054/tube.2000.0263 11463226

[b11-turkjbiol-46-1-95] Hansson M Nygren PA Stahl S 2000 Design and production of recombinant subunit vaccines Biotechnology and Applied Biochemistry 32 2 95 107 10.1042/BA20000034 11001870

[b12-turkjbiol-46-1-95] Hillas PJ del Alba FS Oyarzabal J Wilks A de Montellano PRO 2000 The AhpC and AhpD antioxidant defense system of *Mycobacterium tuberculosis* Journal of Biological Chemistry 275 25 18801 18809 10.1074/jbc.M001001200 10766746

[b13-turkjbiol-46-1-95] Ibrahim El-SE Gamal WM Hassan AI Mahdy SEl-D Hegazy AZ 2015 Comparative study on the immunopotentiator effect of ISA 201, ISA 61, ISA 50, ISA 206 used in trivalent foot and mouth disease vaccine Veterinary World 8 10 1189 1198 10.14202/vetworld.2015.1189-1198 27047016 PMC4774654

[b14-turkjbiol-46-1-95] Galipeau Y Greig M Liu G Driedger M Langlois MA 2020 Humoral Responses and Serological Assays in SARS-CoV-2 Infections Frontiers in Immunology 11 610688 10.3389/fimmu.2020.610688 33391281 PMC7775512

[b15-turkjbiol-46-1-95] SG Iz Sağlam Metiner P Kımız I Kayalı Ç Deliloğlu Gürhan SI 2018 Polyclonal antibody production against hapten-structured KDN molecule by using different adjuvants alternative to freund’s adjuvant European Journal of Therapeutics 24 2 106 111 10.5152/EurJTher.2018.400

[b16-turkjbiol-46-1-95] Jang SI Lillehoj HS Lee SH Lee KW Park MS 2010 Immunoenhancing effects of Montanide ISA oil-based adjuvants on recombinant coccidia antigen vaccination against Eimeria acervulina infection Veterinary Parasitology 172 221 228 10.1016/j.vetpar.2010.04.042 20541870

[b17-turkjbiol-46-1-95] Kanipe C Palmer MV 2020 *Mycobacterium bovis* and you: A comprehensive look at the bacteria, its similarities to *Mycobacterium tuberculosis*, and its relationship with human disease Tuberculosis 125 10 2006 10.1016/j.tube.2020.102006 33032093

[b18-turkjbiol-46-1-95] Kaufmann SHE Parida SK 2008 Tuberculosis in Africa: learning from pathogenesis for biomarker identification Cell Host & Microbe 4 3 219 228 10.1016/j.chom.2008.08.002 18779048

[b19-turkjbiol-46-1-95] Khorasani A Madadgar O Soleimanjahi H Keyvanfar H Mahravani H 2016 Evaluation of the efficacy of a new oil-based adjuvant ISA 61 VG FMD vaccine as a potential vaccine for cattle Iranian Journal of Veterinary Research 17 1 8 12 27656222 PMC4898013

[b20-turkjbiol-46-1-95] Laemmli UK 1970 Cleavage of structural proteins during the assembly of the head of bacteriophage T4 Nature 227 5259 680 685 10.1038/227680a0 5432063

[b21-turkjbiol-46-1-95] Leroux-Roels G 2010 Unmet needs in modern vaccinology: Adjuvants to improve the immune response Vaccine 28 C25 C36 10.1016/j.vaccine.2010.07.021 20713254

[b22-turkjbiol-46-1-95] McCutcheon SR Chiu KL Lewis DD Tan C 2018 CRISPR-Cas Expands Dynamic Range of Gene Expression From T7RNAP Promoters Biotechnology Journal 13 5 e1700167 10.1002/biot.201700167 29149479

[b23-turkjbiol-46-1-95] Michel AL Müller B van Helden PD 2010 *Mycobacterium bovis* at the animal–human interface: A problem, or not? Veterinary Microbiology 140 3–4 371 381 10.1016/j.vetmic.2009.08.029 19773134

[b24-turkjbiol-46-1-95] Okay S Özcengiz E Gürsel I Özcengiz G 2012 Immunogenicity and protective efficacy of the recombinant Pasteurella lipoprotein E and outer membrane protein H from Pasteurella multocida A: 3 in mice Research in Veterinary Science 93 3 1261 1265 10.1016/j.rvsc.2012.05.011 22727197

[b25-turkjbiol-46-1-95] O’Riordan AA Morales VA Mulligan L Faheem N Windle HJ 2012 Alkyl hydroperoxide reductase: A candidate Helicobacter pylori vaccine Vaccine 30 26 3876 3884 10.1016/j.vaccine.2012.04.002 22512976

[b26-turkjbiol-46-1-95] Rodriguez-Campos S Smith NH Boniotti MB Aranaz A 2014 Overview and phylogeny of *Mycobacterium tuberculosis* complex organisms: Implications for diagnostics and legislation of bovine tuberculosis Research in Veterinary Science 97 S5 S19 10.1016/j.rvsc.2014.02.009 24630673

[b27-turkjbiol-46-1-95] Seppic 2017 Montanide^TM^ https://www.seppic.com/sites/seppic/files/2017/02/28/seppic-montanide.pdf accessed 10 February 2021

[b28-turkjbiol-46-1-95] Shah RR Brito LA O’Hagan DT Amiji MM 2015 Emulsions as Vaccine Adjuvants Foged C Rades T Perrie Y Hook S Subunit Vaccine Delivery Springer New York 59 76

[b29-turkjbiol-46-1-95] Sunder S Lanotte P Godreuil S Martin C Boschiroli M 2009 Human-to-human transmission of tuberculosis caused by *Mycobacterium bovis* in immunocompetent patients Journal of Clinical Microbiology 47 4 1249 1251 10.1128/JCM.02042-08 19171683 PMC2668338

[b30-turkjbiol-46-1-95] Towbin H Staehelin T Gordon J 1979 Electrophoretic transfer of proteins from polyacrylamide gels to nitrocellulose sheets: procedure and some applications Proceedings of the National Academy of Sciences 76 9 4350 4354 10.1073/pnas.76.9.4350 PMC411572 388439

[b31-turkjbiol-46-1-95] Valdés I Lazo L Hermida L Guillén G Gil L 2019 Can Complementary Prime-Boost Immunization Strategies Be an Alternative and Promising Vaccine Approach Against Dengue Virus? Frontiers in Immunology 10 1956 10.3389/fimmu.2019.01956 PMC6718459 31507591

[b32-turkjbiol-46-1-95] Verma H Nagar S Vohra S Pandey S Lal D 2021 Genome analyses of 174 strains of *Mycobacterium tuberculosis* provide insight into the evolution of drug resistance and reveal potential drug targets Microbial Genomics 7 3 000542 10.1099/mgen.0.000542 PMC8190606 33750515

[b33-turkjbiol-46-1-95] Whitlow E Mustafa AS Hanif SNM 2020 An overview of the development of new vaccines for tuberculosis Vaccines 8 4 586 10.3390/vaccines8040586 33027958 PMC7712106

[b34-turkjbiol-46-1-95] Wilson T de Lisle GW Marcinkeviciene JA Blanchard JS Collins DM 1998 Antisense RNA to ahpC, an oxidative stress defence gene involved in isoniazid resistance, indicates that AhpC of *Mycobacterium bovis* has virulence properties Microbiology 144 10 2687 2695 10.1099/00221287144-10-2687 9802010

[b35-turkjbiol-46-1-95] Wilson TM Collins DM 1996 ahpC, a gene involved in isoniazid resistance of the *Mycobacterium tuberculosis* complex Molecular Microbiology 19 5 1025 1034 10.1046/j.1365-2958.1996.449980.x 8830260

[b36-turkjbiol-46-1-95] Wong CF Shin J Manimekalai MSS Saw WG Yin Z 2017 AhpC of the mycobacterial antioxidant defense system and its interaction with its reducing partner Thioredoxin-C Scientific Reports 7 5159 10.1038/s41598-017-05354-5 28698569 PMC5505994

[b37-turkjbiol-46-1-95] World Health Organization 2020 Global Tuberculosis Report 2020 Geneva World Health Organization Licence: CC BY-NC-SA 30 IGO

[b38-turkjbiol-46-1-95] Yang E Gu J Wang F Wang H Shen H 2016 Recombinant BCG prime and PPE protein boost provides potent protection against acute *Mycobacterium tuberculosis* infection in mice Microbial Pathogenesis 93 1 7 26792673 10.1016/j.micpath.2016.01.006

