# Supplementary figures and images for: Recombinant AhpC antigen from Mycobacterium bovis boosts BCG-primed immunity in mice
Source: Turk J Biol. 2021 Nov 14;46(1):95–104. doi: 10.3906/biy-2108-41 (PMC10393101; doi:10.3906/biy-2108-41)

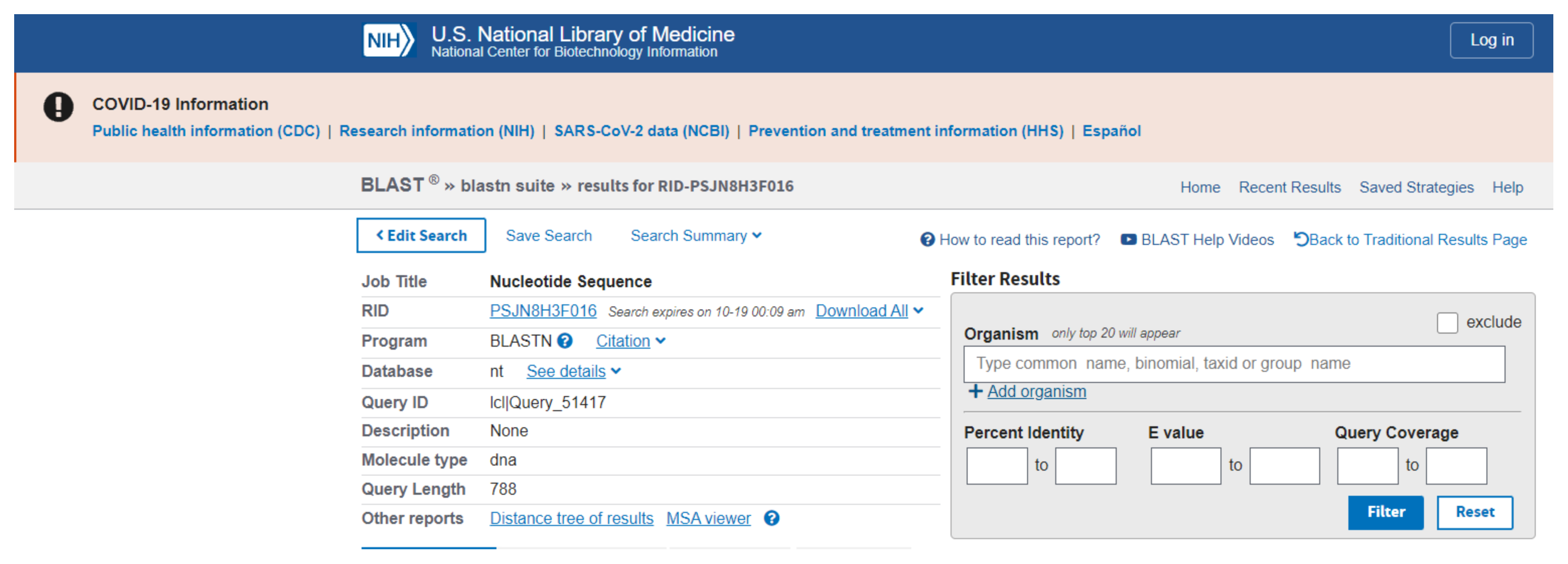

Supplement: Supplementary file 1 [file turkjbiol-46-1-95s1.tif]

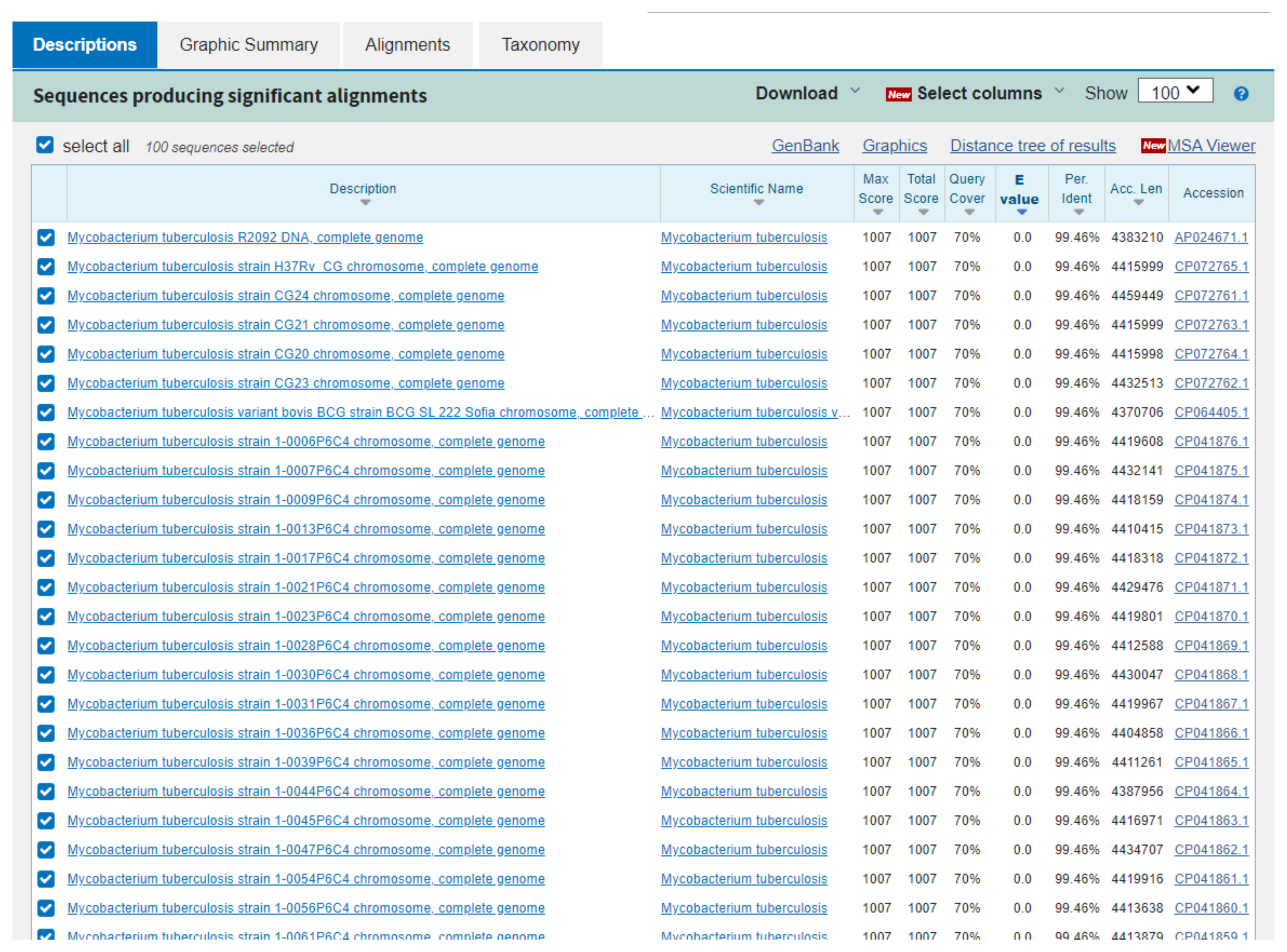

Supplement: Supplementary file 2 [file turkjbiol-46-1-95s2a.tif]

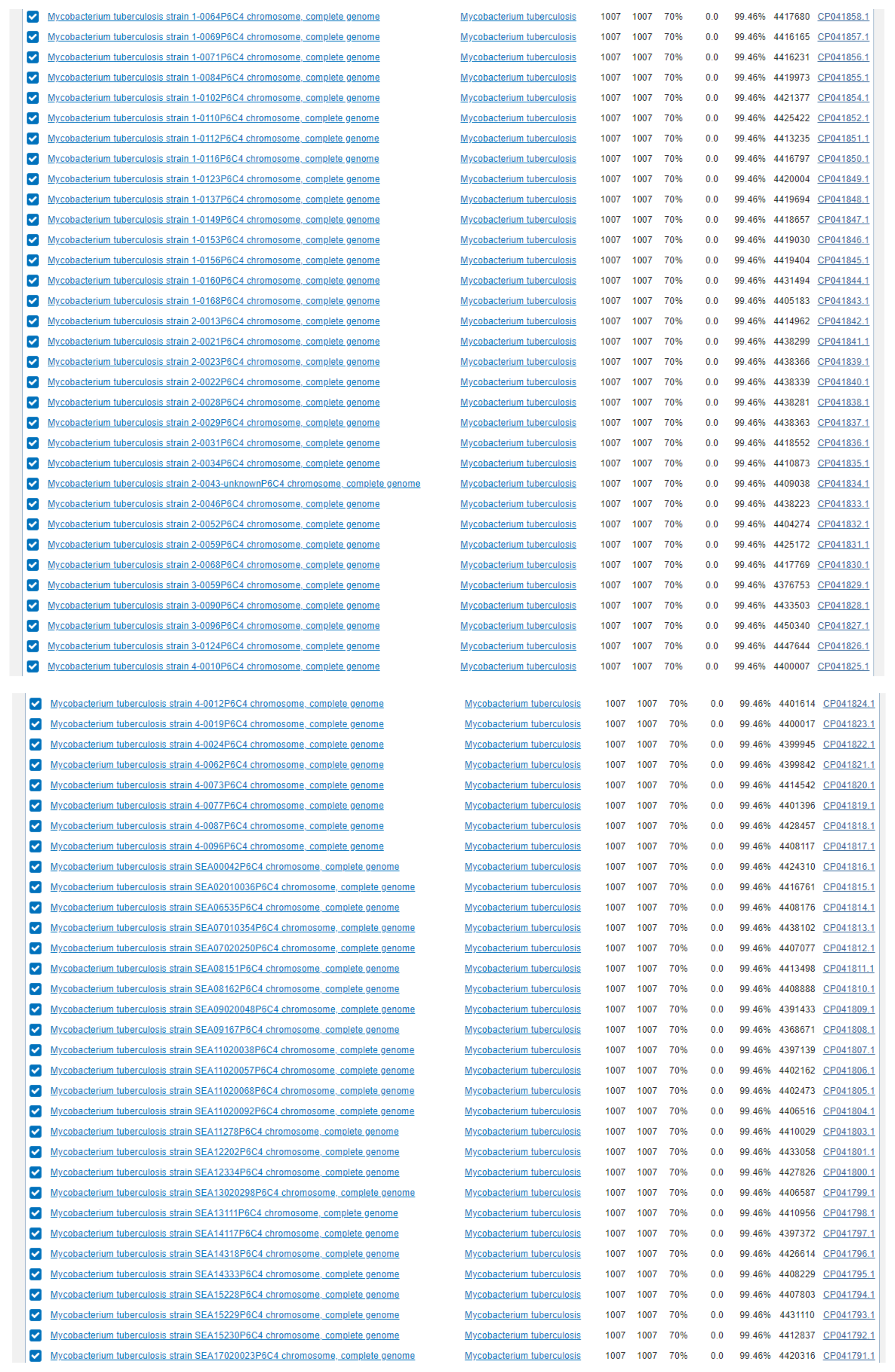

Supplement: Supplementary file 3 [file turkjbiol-46-1-95s2b.tif]

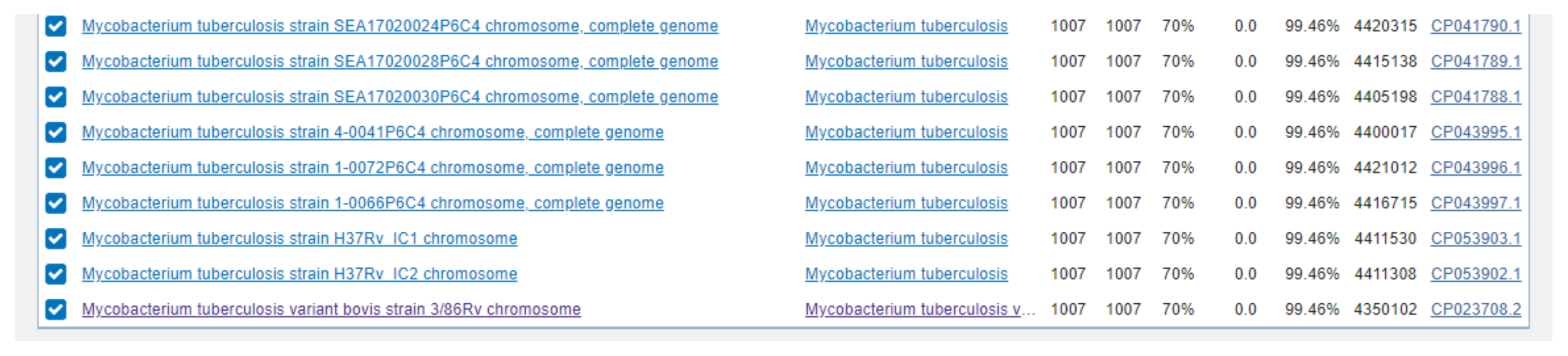

Supplement: Supplementary file 4 [file turkjbiol-46-1-95s2c.tif]
